# Orofacial pain in 1916 patients with early or moderate Parkinson disease

**DOI:** 10.1097/PR9.0000000000000923

**Published:** 2021-04-13

**Authors:** Francis O'Neill, Christopher Kobylecki, Roberto Carrasco, Michele T. Hu, Donald Grosset, Monty Silverdale

**Affiliations:** a Oral Surgery, Institute of Clinical Sciences, University of Liverpool, Liverpool, United Kingdom; b Department of Neurology, Salford Royal NHS Foundation Trust, Manchester, United Kingdom; c Division of Neuroscience & Experimental Psychology, Manchester Academic Health Science Centre, University of Manchester, Manchester, United Kingdom; d Division of Population Health, Health Services Research & Primary Care University of Manchester, Manchester, United Kingdom; e Division of Neurology, Nuffield Department of Clinical Neurosciences, Oxford University, Oxford, United Kingdom; f Department of Neurology, Institute of Neurological Sciences, Queen Elizabeth University Hospital, Glasgow, United Kingdom

**Keywords:** Pain, Parkinson disease, Orofacial, Burning mouth syndrome, Grinding, Chewing

## Abstract

**Introduction:**

Several studies have reported that some types of orofacial pain are more common in patients with Parkinson disease (PD) than the general population.

**Objectives:**

In this study, we aimed to investigate the prevalence of self-reported orofacial pain in a larger group of patients with PD than has been previously studied.

**Methods:**

We analysed data from 1916 participants with PD in a cross-sectional study recruited to the UK Parkinson's Pain Study who had detailed assessments of pain, motor, and nonmotor symptoms. The King's Parkinson's Pain scale was used to quantify different subtypes of pain.

**Results:**

A total of 139 (7.3%) patients reported the presence of some form of orofacial pain. Burning mouth syndrome was reported in 32 (1.7%), whereas chewing pain was found in 38 (2.0%) and grinding pain in 78 (4.0%). Orofacial pain was significantly more common in females (10.4%) than males (5.9%). Multiple logistic regression analysis showed a significant association between orofacial pain and pain severity, neuropathic pain, and oral motor and nonmotor dysfunction.

**Conclusion:**

In our study, population cohort of early patients with PD found prevalence of orofacial pain conditions similar to that in the general population.

## Abstract 2

This article reports the largest epidemiological study of orofacial pain prevalence in patients with Parkinson disease to date.

## 1. Introduction

Sixty to eighty-five percent of people with Parkinson disease (PD) experience chronic pain,^7,45,47^ and it is considered a major nonmotor symptom of PD. Mostly pain symptoms affect large areas of the body, eg, musculoskeletal, dystonic, and radicular pains. Orofacial pain can be divided into odontogenic (ie, from teeth or teeth baring structures) and nonodontogenic pain. In the latter group are conditions such as burning mouth syndrome (BMS), temporomandibular disorders (TMDs), muscular pain related to grinding or chewing, and headaches.^25^ All pain can be classified as nociceptive, neuropathic, or nociplastic.^24^ Patients with PD have been shown to experience each type of pain in 55%, 16%, and 22% of case, respectively.^44^ Burning mouth syndrome is becoming more accepted as a neuropathic pain,^29^ whereas temporomandibular joint pain or TMDs are mostly considered nociceptive pain. However, chronic TMDs may be nociplastic in origin especially when related to other conditions such as fibromyalgia.^31^

An association between orofacial pain and PD has been suggested with several authors reporting that some types of orofacial pain are more common in PD than in the general population.^15,16^ For instance, best estimates of the prevalence of BMS in the general population range between 0.11% and 3.7%.^30^ However, several postal surveys with sizes of around 200 patients with PD have found BMS prevalences of 4.0%,^11^ 9.7% to 14.0%,^15^ and 24.0%.^16^ A study of 178 patients with PD with pain compared with 83 matched controls, using the King's Parkinson's Pain scale (KPPS) found a prevalence of BMS in patients with PD of 5.1%, compared with 1.2% in controls.^38^ Because all patients by definition had pain, this may not have been representative of the wider population with PD. Furthermore, in a recent Dutch study,^52^ patients with PD or parkinsonism self-reported more frequent orofacial pain, TMD pain, and bruxism than healthy controls. However, the absolute frequencies of these symptoms were not recorded. These studies were rather small for epidemiological studies, and a larger study size would help in understanding the true extent of this association and whether it still exists in large sample sizes.

Shared mechanisms may underly pathophysiology in PD and trigeminal pain involving the dopaminergic system. First, PD is characterised by low dopamine levels,^32^ and dopamine is known to have complex effects on the transduction of pain signalling. Low levels of dopamine stimulate D2 receptors and may inhibit pain transmission, whereas higher levels stimulate D1 receptors and may facilitate pain transmission.^1–3,12,49^ Second, electrophysiological studies suggest the involvement of dopamine in the deficient habitation of the trigeminal blink reflex that occurs in BMS^6,20,27,33^ and also in PD.^20,33^ Third, positron emission tomography studies in humans with BMS have demonstrated a reduction in presynaptic striatal dopamine with a consequent increase in D2 receptor binding.^22,28^ Fourth, dopaminergic medications have been used to treat BMS,^17,48^ and finally, polymorphisms in genes involved in dopamine metabolism are a risk factor for TMD pain.^18,19,41^ Taken together, these studies support a role of dopamine dysfunction in at least some patients with facial pain.

In this study, we aimed to understand the prevalence of self-reported orofacial pain in a much larger detailed clinical group of patients with PD than has been hitherto investigated. We further aimed to explore the associations between orofacial pain and overall pain severity, as well as the relationship between orofacial pain and motor dysfunction, specifically regarding areas of oral motor control.

Given the association of trigeminal pain and differential action of D1 and D2 dopamine receptors and the dose dependant activation of those receptors by dopamine, we hypothesised that pain may be more prominent in patients taking higher doses of levodopa.

## 2. Materials and methods

### 2.1. Ethical approval

The study was conducted in accordance with the Declaration of Helsinki and authorized by a UK ethics committee (National Research Ethics Service Committee North West). All patients gave written consent before any study procedures.

### 2.2. Participants

All participants were recruited from one of 2 large UK multicentre longitudinal epidemiological and biomarker studies in PD; those being Tracking Parkinson's^37^ and the Oxford Monument Discovery Study.^35^ The inclusion and exclusion criteria for those studies have been previously published.^35,37^ The pain study was a substudy using the same research nurses, although it was funded and run separately. It was performed at a single occasion at any one of the main study visits.

For the assessment of orofacial pain, pain was assessed using the KPPS, which quantifies pain across 7 different subtype domains. For each subtype, the participant rates severity (0–3) and frequency (0–4), which are then multiplied to create a total score for that subtype of pain. The subtypes include musculoskeletal pain, chronic pain, fluctuation-related pain, nocturnal pain, orofacial pain, discolouration oedema or swelling, and radicular pain.

### 2.3. Specific questions on orofacial pain

Domain 5 on the KPPS includes the following specific orofacial pain questions: Does the patient have BMS? Does the patient experience pain when chewing? Does the patient have pain due to grinding their teeth at night?

Other pain questionnaires also administered were the visual analogue scale for pain severity, the short-form McGill pain questionnaire,^39^ and the Leeds Assessment of Neuropathic Symptoms and Signs (LANSS) scale was used to document the presence of neuropathic pain.^9^ The LANSS scale is validated to detect neuropathic pain and uses an assessment of sensory function (cutaneous allodynia and altered pinprick threshold over the painful area) as well as pain descriptors to classify pain that is likely to be centrally generated. High pain was defined as to have at least one sensory item ≥3 from the McGill questionnaire and significant pain as at least one sensory item = 2.

The Movement Disorder Society Unified Parkinson's Disease Rating Scale (MDS-UPDRS) parts I to IV was assessed in all participants. Levodopa equivalent daily dose (LEDD) was calculated using the method of Tomlinson 2010.^50^ Affective symptoms were using the Leeds Anxiety and Depression Scale (LADS).

### 2.4. Statistical analysis

We calculated the prevalence of orofacial pain groups within this PD cohort. Total percentages of any orofacial pain, BMS, chewing pain, and grinding pain were plotted by gender. Comparison of orofacial pain prevalence by gender was performed by the Fisher exact test. Separate multivariable logistic regression models were used to investigate the factors predicting any orofacial pain, BMS, chewing pain, and grinding pain. Factors were included in the multivariable logistic regression if they were significantly associated in a univariate preliminary analysis or if they were considered clinically relevant. Therefore, factors in the models included KPPS total, LADS, McGill, pain level (high or significant pain), neuropathic pain, dyskinetic pain, UPDRS saliva or drooling, UPDRS speech, UPDRS eating, and UPDRS chewing or swallowing. The models were adjusted for gender (M/F), age (years), LEDD, LADS, smoking status, and diabetes history. Missing data points were treated on a pairwise basis.

The χ^2^ test and Wilcoxon rank test were used to investigate factor differences with any orofacial pain, BMS, chewing pain, and grinding pain. Factors studied were high pain, significant pain, neuropathic pain, and LEDD. Bonferroni correction was applied to minimise the risk of a type I error, and significance was set at *P* ≤ 0.00125 for logistic regression analyses and *P* ≤ 0.00416 for χ^2^ tests and Wilcoxon rank tests. All analyses were performed with Stata/IC 14.0 (StataCorp LP).

## 3. Results

### 3.1. Study population and pain characteristics

In total, 1957 participants were recruited into the UK Parkinson's Pain Study. The mean age of patients in the study was 68.0 years (±9.5 years, SD); 1272 (65.0%) were male, and 685 (35%) were female. The mean disease duration was 3.0 years (±2.1 years). Forty-one (2.0%) patients were excluded because of missing orofacial pain data; overall percentages were calculated as a proportion of the remaining 1916 participants. Of these 1916 participants, an additional 41 (2.1%) had one or more orofacial pain data missing at random, and these missing data were treated on a pairwise basis. More detailed analysis of this cohort is published elsewhere.^47^ Here, we focus on orofacial pain.

### 3.2. Orofacial pain prevalence

**Figure 1.**
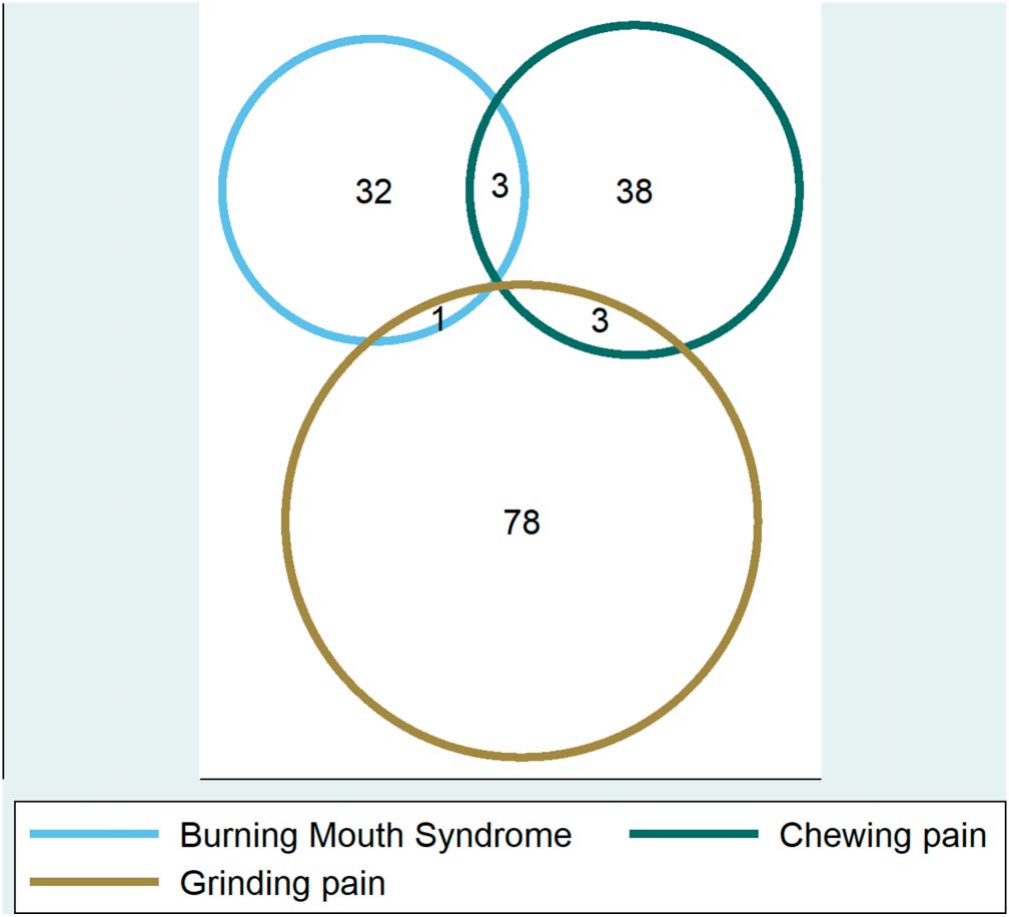
Venn diagram indicating very small proportion of overlap between groups.

**Figure 2.**
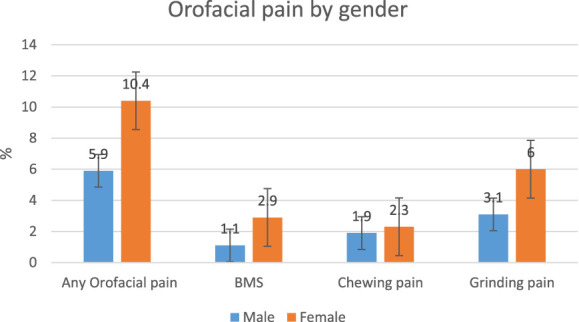
Percentage of total gender within orofacial pain groups.

A total of 139 (7.3%) of patients reported the presence of some form of orofacial pain, as shown in Figure 1 and Table 1. Orofacial pain was reported in 69 (10.4%) of female participants and 70 (5.9%) of males participants. Thirty-two (1.7%) patients reported the presence of BMS with a female-to-male ratio of 3:1. Thirty-eight (2.0%) patients declared (2.3:1.9 F:M ratio) pain when chewing and 78 (4.0%) (2:1 F:M ratio) reported grinding pain, as shown in Figure 2. The mean age between those without orofacial pain and those with any orofacial pain, chewing pain, or BMS was not significantly different. Those who reported grinding pain however were significantly younger (65 years old, SD 10) than those without facial pain (68.1 years old, SD 9.4).

**Table 1:** Total prevalence of orofacial pain groups of 1916 patients within Parkinson disease cohort.

	Any orofacial pain	Burning mouth syndrome	Chewing pain	Grinding pain
No (%)	1736 (90.7)	1859 (97)	1858 (97)	1817 (95)
Yes (%)	139 (7.3)	32 (1.7)	38 (2)	78 (4)
Missing (%)	41 (2)	25 (1.3)	20 (1)	21 (1)

### 3.3. Association with dyskinetic pain

Patients who reported burning mouth symptoms or pain on grinding showed significantly higher mean dyskinetic pain scores on the KPPS (no BMS 0.36 [SD 1.5] vs BMS 1.55 [SD 3.1] and no grinding pain 0.35 [SD 1.45] vs grinding pain 0.77 [SD 2.45]). Patients reporting chewing pain had similar dyskinetic pain scores. When adjusted for gender, age, LADS, LEDD, smoking status, and diabetes history, patients with dyskinetic pain were slightly more likely to experience symptoms of burning mouth (odds ratio 1.17, *P* = 0.007); however, this was not statistically significant after Bonferroni correction, refer to Table 2.

**Table 2:** Logistic regression for each variable, adjusted by gender (M/F), age (years), levodopa equivalent daily dose, Leeds Anxiety and Depression Scale, smoking status, and diabetes.

	Any orofacial pain			Burning mouth syndrome			Chewing pain			Grinding pain		
	OR	*P*	95% CI	OR	*P*	95% CI	OR	*P*	95% CI	OR	*P*	95% CI
KPPS total	1.05	<0.0001*	1.04-1.06	1.03	0.003*	1.01-1.05	1.05	<0.0001*	1.02-1.07	1.02	<0.0001*	1.01-1.04
Full McGill	1.07	<0.0001*	1.04-1.09	1.04	0.13	0.99-1.1	1.09	<0.0001*	1.04-1.13	1.05	0.001*	1.02-1.08
Significant pain	2.3	0.004	1.3-4.2	2.1	0.2	0.7-6.7	3.2	0.047	1.01-9.9	2.3	0.034	1.06-5.1
High pain	2.7	0.001*	1.5-4.9	1.2	0.75	0.3-4.2	3.8	0.025	1.2-12.05	3.0	0.006	1.37-6.5
Neuropathic pain	3	<0.0001*	1.8-4.95	2.9	0.03	1.1-7.4	2.1	0.15	0.75-5.8	3.1	<0.0001*	1.7-5.7
Dyskinetic pain	1.06	0.2	0.97-1.16	1.17	0.007	1.03-1.34	0.96	0.8	0.76-1.2	1.01	0.9	0.89-1.13
UPDRS saliva/drooling	1.13	0.14	0.96-1.3	1.03	0.9	0.7-1.4	1.1	0.5	0.8-1.5	1.17	0.12	0.95-1.44
UPDRS speech	1.3	0.006	1.09-1.6	0.8	0.4	0.5-1.3	1.4	0.07	0.97-2.1	1.54	0.001*	1.2-2
UPDRS eating	1.4	0.016	1.06-1.8	1.3	0.3	0.76-2.2	1.7	0.03	1.05-2.75	1.3	0.12	0.9-1,8
UPDRS chewing/swallowing	1.9	<0.0001*	1.4-2.4	1.9	0.006	1.2-3.1	1.6	0.06	0.97-2.6	1.7	0.001*	1.23-2.3
NMSS swallowing	1.2	0.003*	1.06-1.4	1.3	0.02*	1.03-1.5	1.3	0.007	1.08-1.7	1.06	0.4	0.9-1.25

* *P* value ≤ 0.00125 was considered statistically significant.

CI, confidence interval; KPPS, King's Parkinson's Pain Scale; NMSS, Non-Motor Symptoms Scale; OR, odds ratio; UPDRS, Unified Parkinson's Disease Rating Scale.

### 3.4. Association with neuropathic pain

The presence of any orofacial pain, BMS, and grinding pain was all significantly related to the presence of other bodily neuropathic pain signs and symptoms as detected on the LANSS scale. In total, 25.5% of patients reporting any orofacial pain had concomitant neuropathic pain compared with only 6.7% of PD without orofacial pain. Within pain subgroups, 34.4% patients reporting BMS had concomitant neuropathic pain compared with 7.8% without and 28.2% of patients with grinding pain compared with 7.4% without (*P* = 0.0001). Although 15.8% of patients with chewing pain had features of neuropathic pain compared with 8.1% without, this did not reach statistical significance (*P* = 0.09). When adjusted for gender, age, LADS, LEDD, smoking status, and diabetes history, patients with neuropathic pain were significantly more likely to have any orofacial pain or grinding pain (Table 2).

### 3.5. Relation to oral motor dysfunction

When subdomains within the MDS-UPDRS were compared, we found higher levels of oral disability for eating, chewing or swallowing, and speech in the orofacial pain group. Concerning pain subtypes, chewing or swallowing was more impaired in patients with BMS and grinding pain, and speech was more impaired in those with grinding pain. By contrast, overall motor dysfunction as measured with the total MDS-UPDRS-III score was not associated with orofacial pain. When adjusted for gender, age, LADS, LEDD, smoking status, and diabetes history, patients with chewing or swallowing deficits as measured by the UPDRS were more likely to have any orofacial pain and grinding pain.

### 3.6. Association with levodopa dosage in patients with Parkinson disease

Patients reporting BMS or pain on grinding used a significantly higher daily dose of Parkinson medication. The median LEDD in those without BMS was 400 mg (interquartile range 250–550) vs BMS 465 mg (300–660, *P* = 0.01) and no grinding pain 400 mg (250–550) vs grinding pain 462.5 mg (300–669, *P* = 0.009). However, after Bonferroni correction, these differences were not significant. Patients with pain on chewing did not show any significant difference in LEDD, refer to Table 3.

**Table 3:** Correlation between orofacial pain groups and high pain levels, neuropathic pain presence, and total levodopa equivalent daily dose.

Any orofacial pain	No (n = 1736)	Yes (n = 139)	*P*
High pain, n (%)	485 (27.9)	65 (46.7)	<0.0001*
Neuropathic pain, n (%)	117 (6.7)	36 (25.5)	<0.0001*
LEDD total, median (IQR)	400 (250–450)	400 (300–615)	0.01*

BMS	No (n = 1859)	Yes (n = 32)	*P* Value
High pain, n (%)	536 (28.8)	13 (40.6)	0.14
Neuropathic pain, n (%)	146 (7.8)	11 (34.4)	<0.0001*
LEDD total, median (IQR)	400 (250–550)	465 (300–660)	0.01*

Chewing pain	No (n = 1858)	Yes (n = 38)	*P* Value
High pain, n (%)	531 (28.5)	17 (44.7)	0.029
Neuropathic pain, n (%)	151 (8.1)	6 (15.8)	0.09
LEDD total, median (IQR)	400 (260–550)	347.5 (200–480)	0.2

Grinding pain	No (n = 1817)	Yes (n = 78)	*P* Value
High pain, n (%)	511 (28.1)	37 (47.4)	<0.0001*
Neuropathic pain, n (%)	135 (7.4)	22 (28.2)	<0.0001*
LEDD total, median (IQR)	400 (250–550)	462.5 (300–669.2)	0.009*

BMS, burning mouth syndrome; IQR; interquartile range; LEDD, levodopa equivalent daily dose.

### 3.7. Disease duration

There was no difference in “time since diagnosis” between those with and without “any orofacial pain,” chewing pain, or grinding pain. However, disease duration was longer in those with BMS (5.8 years ± 7.3, SD) compared with those without BMS (2.9 years ± 1.9 SD, *P* = 0.03).

## 4. Discussion

We report a large study of patients with early–moderate PD and report an overall prevalence of orofacial pain of 7.3%, with associations between orofacial pain and oral motor disability and higher pain scores in general. The demographics of this cohort are comparable with those of previous studies with a similar male or female distribution,^11,16,38^ although our participants are at an earlier stage of PD.

Although 85% of the cohort experience some form of pain,^53^ just more than 7% report orofacial pain. Contrary to some previous evidence, the prevalence of orofacial pain subgroups is remarkably similar to that in the general population. The frequency of BMS in this group is at the lower end of best estimates from the general population.^30^ Kohorst et al.^34^ have shown a prevalence of BMS in the general population as low as 0.11%. This study was conducted in Olmsted County in Minnesota, USA, which has a relatively young population with a total average age of 37 years. The population sample in our study is much older with an average age of 68 years and therefore may not be directly comparable. Other studies of BMS prevalence have been undertaken in other settings with a more similar age profile, for instance, a telephone survey of volunteers in Florida (average age around 65 years) that showed a prevalence of 1.7%.^46^ Furthermore, our study examines the prevalence of burning symptoms in a patient group that already has another comorbid condition, ie, Parkinson disease and therefore likely shift demographics from that of the general population. Comparing with another study that examines prevalence in a group with a comorbidity, Moore et al.^42^ looked at BMS in 371 adults with type 1 diabetes and found a prevalence of 2.1%. An age-matched population without PD may be a useful comparator. This result is certainly lower than previous studies suggesting that BMS is more common in patients with PD than healthy controls.^16,38^

Some previous studies were restricted to patients with PD who reported pain^38^ so may have overestimated the overall prevalence of pain subtypes. Although 85% of patients in this sample presented with pain, differences due to overestimation would likely be modest. The prevalence of painful TMD-related pains in the general population as a whole is between 2.0% and 7.0%,^26^ and both the frequency of chewing pain (2.0%) and grinding pain (4.0%) in this study are within this range. Prevalence may be subject to participant selection or recruitment bias, although this was a large study of patients not specifically selected because of pain characteristics. The fact that this is a sample of relatively early stage PD may limit the generalizability of our findings. It is possible that orofacial pain frequency and severity increases with PD duration and this may emerge from follow-up that is ongoing. In support of this, participants with BMS had a longer disease duration than those without. Mechanisms potentially responsible for this are unclear but putative reasons for example could include phenomena such as cobalamin deficiency with advanced PD duration.^51^ However, we did not see any correlation between “any orofacial pain,” chewing pain, or grinding pain and disease duration in our cohort.

We found that orofacial pain was associated with both swallowing and speech problems, but not overall parkinsonian disability. Axial symptoms such as dysphagia in PD generally show a minimal response to dopaminergic medications^40^ suggesting other mechanisms. Parkinson disease–related alpha-synuclein pathology has been identified in sensory nerve terminals in the oropharynx of patients with PD compared with controls.^43^ Such peripheral involvement could be a common pathway between oral dysfunction and orofacial pain in PD. It is an open question whether treatment to improve orofacial pain might therefore improve both speech and swallowing issues in PD, but our data suggest that this could be a promising line of investigation for these disabling refractory PD symptoms. Indeed, rehabilitative trials for oral dysfunction in PD have recently been reported,^5^ and their effects on orofacial pain would be of interest. It can also be seen, in this cohort, that not all of the 3 subtypes of orofacial pain recorded have the same relationships with other disease-related symptoms. Although there is some crossover of patients with these different types of facial pain, there were no patients who experienced all 3 simultaneously.

As hypothesised, the study does show an association between orofacial pain symptoms and LEDD with those reporting “any orofacial pain” and “grinding pain” taking a significantly higher daily dosage of medication than those without these orofacial pain types. The overall LEDD taken by patients in this cohort was less than in previous studies, most likely as this is a cohort with earlier PD. Xerostomia has been associated with levodopa use in previous studies^15^ and represents a potential pathway for increased pain as well as other oral dysfunctions, such as dysphagia. Similarly, we note a correlation with dyskinetic pain and BMS. Dyskinesia is a marker of overstimulation of dopamine receptors,^13^ further supporting the link between high levels of dopamine and orofacial pain.^21,22,36^

Taken together, the 2 points above may suggest that those patients with worse overall symptoms tend to take higher doses of medication and potentially this tips the balance at striatal D1 and D2 dopamine receptors towards facilitation of pain.

Neuropathic pain is defined as pain caused by damage or disease affecting the somatosensory system^23^ and involves central nervous system sensitisation. Interestingly, we also demonstrate an association between orofacial pain and neuropathic pain (as measured with the LANSS scale, which is validated to identify neuropathic pain^9^ and similar in sensitivity and specificity to the Douleur Neuropathic 4 and painDETECT screening tools).^4,8,10^ Our data therefore back up previous studies supporting the role of central neuropathic mechanisms in the generation of some orofacial pains.^14^

We acknowledge that there are several limitations to our study which mean that the results must be interpreted cautiously. The study population was mainly of early and moderate cases and detailed data on other comorbidities and analgesic use were not available, so the results are not necessarily generalisable.

The pain conditions of BMS, pain on grinding, and pain on chewing were not independently assessed to ensure accurate attribution to origin of pain, and therefore, we have had to accept self-reported data that may include self-diagnosis. The question of pain on chewing is rather nonspecific and does not exclude simple dental pain due to tooth decay or gum disease. However, given the much higher prevalence of both of these conditions in the general population either there is a very small representation in this cohort or patients are able to discern between these sources of pain and explicit pain on chewing. Bonferroni correction was applied to minimise type I error, and as a result, the significance levels were very conservative and may increase the probability of producing false negatives. As with any large study, there are some missing data that could have affected the results.

## 5. Conclusions

We believe this to be the largest study of orofacial pain in patients with PD to date. The cohort is well characterised, and although the measurements collected are self-reports, the questionnaires used are well-validated instruments used in Parkinson-related studies internationally. In our study population cohort of patients with early PD, we found prevalence of orofacial pain conditions similar to that in the general population. The potential relationship between orofacial pain and markers of oral disability in PD suggests a need for better evidence for orofacial pain management, given the fact that such parkinsonian features may be relatively refractory to antiparkinsonian medication.

## Disclosure

C. Kobylecki has received grants from Parkinson's United Kingdom and the Michael J Fox Foundation; speaker fees from Britannia and Bial Pharma; and support to attend international meetings from Abbvie. M.T. Hu received funding or grant support from Parkinson's United Kingdom, Oxford NIHR BRC, University of Oxford, CPT, Lab10X, NIHR, Michael J Fox Foundation, H2020 European Union, GE Healthcare, and the PSP Association. She also received payment for Advisory Board attendance or consultancy for Biogen, Roche, CuraSen Therapeutics, Evidera, and Manus Neurodynamica. M.T. Hu is a co-applicant on a patent application related to smartphone predictions in Parkinson disease (PCT/GB2019/052522) patent pending. D. Grosset has received grants from Parkinson's United Kingdom, Michael's Movers, and the Neurosciences Foundation; speaker fees from Vectura plc and Merz Pharma; and consultancy fees from the Glasgow Memory Clinic. M. Silverdale has received grant funding from Parkinson's United Kingdom and Michael J Fox Foundation; meeting honoraria from UCB as well as conference expenses from Bial, Abbvie, and Medtronic. The remaining authors have no conflicts of interest to declare.

This work was funded by Parkinson's United Kingdom (grant number K1301). The funding source had no other involvement in the study.
